# A method for recognizing positive and negative electrodes of buzzer circuit board based on machine vision

**DOI:** 10.1371/journal.pone.0346866

**Published:** 2026-04-15

**Authors:** Xiaoyang Liu, Cheng Wang, Xupeng Huang, Chenxin Sun, Rongjin Zhu, Chongyang Hu, Qianheng Ding

**Affiliations:** Faculty of Automation, Huai’an University, Huai’an, China; UNICAMP, University of Campinas, BRAZIL

## Abstract

In order to automate the soldering of coils to circuit boards in buzzers, this study proposes a method for recognizing positive and negative electrodes of buzzer circuit board based on machine vision. The method distinguishes and locates the positive and negative electrode of the circuit boards, which facilitates subsequent soldering operations. Since buzzer circuit boards are left-right symmetric and the positive and negative solder pads are highly similar in morphology, it is difficult to distinguish between the positive and negative electrodes based on visual features. The method first employs the color difference operator *R-G-B* to extract the color feature map of the circuit board. Next, algorithms such as filtering, threshold segmentation and contour detection are employed to extract the circuit board contours, and the rotated minimum bounding rectangle of each circuit board is obtained to achieve precise locating. Then, according to the up-down asymmetric geometric characteristics of the circuit boards, the positive and negative electrode recognition problem is converted into a simple geometric analysis problem. According to the current orientation of the circuit board, it is categorized into eight cases, and corresponding calculation formulas of positive and negative position are designed, effectively distinguishing and locating the positive and negative electrodes of the circuit board. Experimental results demonstrate high recognition accuracy of the method: The error in extracting the minimum bounding rectangle is only single-digit(zero to ten) pixel values. Finally, the average relative error in recognizing the positive and negative electrode positions is less than 2%. Tests confirm that the proposed method achieves robust recognition and locating performance, showing promising potential for application in automatic soldering equipment to realize automated soldering of buzzer circuit boards.

## 1. Introduction

Buzzers, as electronic components, are widely used in electronic products such as computers, printers, alarms, timers, electronic toys. Their primary function is to generate sustained and sharp sound signals for alerts or warnings. Buzzers are categorized into two types based on their driving methods: active buzzers and passive buzzers. Active buzzers incorporate an internal oscillation circuit, so they can work as long as the DC power is supplied, without additional driving circuits. Passive buzzers, lacking an internal oscillation circuit, must be driven by an external square wave signal to work. When connecting to an external DC power supply, it is necessary to distinguish the polarities. The positive and negative electrodes of the buzzer circuit board should be matched to the corresponding positive and negative poles of the power supply. Similarly, when connecting to an external oscillation signal, it is necessary to distinguish between high and low frequencies. The positive and negative electrodes of the buzzer circuit board should be matched to the corresponding high and low frequencies of the oscillation signal. Therefore, the positive and negative electrode recognition of the buzzer circuit board is of great significance. It enables correct matching when the buzzer circuit board is connected to the external components, making the device work normally. The soldering for buzzer circuit boards and oscillation coils typically relies on manual labor, where operators must visually recognize the positive and negative solder pads during the process. However, manual soldering suffers from slow speed, inconsistent quality, and frequent issues such as soldering errors, uneven solder joints, and position deviations. In recent years, automated soldering equipment integrated with machine vision has progressively replaced manual soldering. This technology not only enhances soldering efficiency and quality but also improves soldering accuracy.

With the development of artificial intelligence technology, machine vision has been extensively applied across various industries [1]. In the food industry, machine vision is primarily used for food quality inspection [2–4] and packaging defect inspection [5–7]. In the machinery industry, it is mainly employed for precision measurement [8–10], workpiece positioning, and industrial robot navigation [11–13]. In agriculture, machine vision has a wide range of applications, including visual navigation, non-destructive testing, precise positioning, pest and disease identification and detection, growth monitoring, feature recognition or judgment, and information collection or calculation [14].

In the electronics industry, machine vision is significantly used for the inspection of circuit boards, primarily for circuit board recognition, positioning, and defect detection. Zhou et al. [15] developed a Mark point positioning approach using template matching and improved Hough transform, and experimental results demonstrated high efficiency of the former and the strong anti-interference capability of the latter. Xia [16] employed connected component analysis to preliminarily determine the position of circuit board positioning holes, then applied two-dimensional wavelet transform to accurately identify the positioning holes, demonstrating high stability and reliability. Bao et al. [17] proposed a printed circuit board Mark point positioning method based on the IBBS-SIFT (Improved Best-Buddies Similarity Invariant Feature Transform) algorithm, which improved and integrated the IBBS and SIFT algorithms, boosting the efficiency and precision of Mark point positioning. Gao [18] used a sub-pixel least squares fitting algorithm to locate Mark points on printed circuit boards, which is more accurate and robust than the normalized template matching algorithm. For the identification of fiducial marks or positioning holes on circuit boards, the above methods demonstrate effective capabilities. For the identification of solder joints on circuit boards, the following methods also perform well. Zhang [19] integrated the SIFT algorithm with an edge detection algorithm to realize recognition and data extraction of solder joints on circuit boards, and experiments proved that this algorithm has minimal error. Chen [20] designed a PCB solder joint positioning method based on image feature point detection and matching using the SURF algorithm, and it calculated the homography matrix between the detected PCB image and the template image to achieve accurate positioning of the solder joint coordinates. Fan [21] proposed an improved YOLOv4 algorithm for identification and localization of circuit board solder joints, which improved detection speed while ensuring detection accuracy. Pan et al. [22] used red, green, and blue light sources at different angles and assessed solder joint positions with circular fitting similarity calculations, achieving high positioning accuracy.

For circuit board defect detection, applications of machine vision technology include image processing-based methods and machine learning-based methods [23]. Zhong et al. [24] achieved defect recognition and classification across varying backgrounds in flexible circuit board images by calculating pixel background feature probabilities in edge regions between backgrounds and defects. Chang et al. [25] first optimized the Otsu thresholding technique with PSO to separate defect regions from the background, then combined the FLANN (Fast Library for Approximate Nearest Neighbors) algorithm with RANSAC (Random Sample Consensus) for image alignment, finally detected defects through image subtraction and morphological processing. Luo et al. [26] proposed an optimal PCB defect detection method based on rotation angles, showing high effectiveness, fast speed, and fewer model parameters required. The traditional image processing-based methods mentioned above offer fast detection speed, but their adaptability is generally limited, and their effectiveness for complex defects is constrained. Therefore, some scholars have proposed methods based on machine learning and deep learning, which are more suitable for handling complex defect detection tasks. Zheng et al. [27] proposed an improved speeded-up robust features (SURF) and random forest (RF) algorithm, where the enhanced SURF algorithm first boosted the information density of feature data, and data preprocessing subsequently improved the RF algorithm, significantly enhancing accuracy and reducing training time. Park et al. [28] designed a multi-label classification network for images of various sizes (MarsNet) and horizontal vertical pooling (HVP), employing a multi-label scoring module and a threshold estimation module to detect solder paste defects on circuit boards. Lin et al. [29] designed an edge multi-scale reverse attention network (EMARS-Net), showing great potential for detecting tiny and low-contrast defects on circuit boards. Wang et al. [30] proposed a method based on pseudo-inverse transform and improved YOLOv5, which enhanced the quality of image processing and improved the accuracy of detecting defects in printed circuit boards. Wei et al. [31] proposed an improved PCB-YOLO model based on YOLOv8n, introducing the CRSCC module and FFCA attention module while developing the WIPIoU loss function, thereby achieving outstanding performance in PCB defect detection tasks. Niaz et al. [32] designed a hybrid framework that combined Vision Transformers with a Mamba-inspired attention mechanism (ViT-Mamba) for global feature extraction and precise defect segmentation, achieving good detection results on a public circuit board defect dataset. Lan et al. [33] proposed a method named CM-YOLO for circuit board defect detection based on the fusion of RGB images and depth images using YOLO11, and extensive experiments demonstrated that this method achieves very high average precision. Xu et al. [34] developed the RetC3K2 module and a multi-branch auxiliary neck network on the foundation of YOLO11n, which improved detection accuracy while ensuring detection speed in circuit board defect detection.

**Fig 1:**
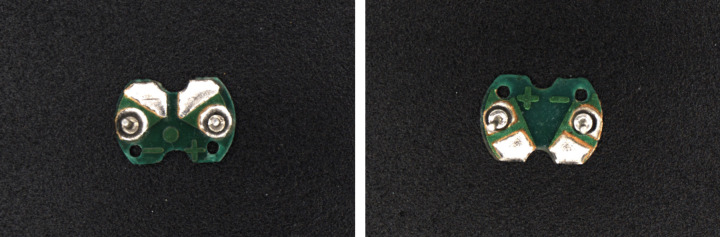
Image of the buzzer circuit board.

The above research studies demonstrate the application of machine vision in the electronics industry, especially in circuit board recognition, positioning, and defect detection, using various methods from traditional image processing to modern machine learning. These research studies hold certain referential significance for this paper. At present, although there are many types of automatic soldering equipment, most of them can only recognize general circuit board solder joints and cannot effectively identify and distinguish the positive and negative solder joints of buzzer circuit boards. Compared with the recognition of general solder joints demonstrated in the above research studies, recognizing the positive and negative solder pads on buzzer circuit boards is more challenging. Because buzzer circuit boards are left-right symmetric and the positive and negative solder pads are highly similar in morphology (as shown in Fig 1), it is difficult to distinguish only based on visual features. Therefore, this study proposes a method for recognizing positive and negative electrodes of buzzer circuit board based on machine vision. The method first employs the color difference operator *R-G-B* to extract the color feature map of the circuit board. Next, algorithms such as filtering, threshold segmentation and contour detection are employed to extract the circuit board contours, and the rotated minimum bounding rectangle of each circuit board is obtained to achieve precise locating. Then, according to the up-down asymmetric geometric characteristics of the circuit boards, the positive and negative electrode recognition problem is converted into a simple geometric analysis problem. According to the current orientation of the circuit board, it is categorized into eight cases, and corresponding calculation formulas of positive and negative position are designed, effectively distinguishing and locating the positive and negative electrodes of the circuit board. This method fully utilizes the geometric characteristics of the circuit board, achieving high recognition accuracy and robust performance. It is expected to be applied to automatic soldering equipment to achieve automated soldering of buzzer circuit boards.

Currently, deep learning algorithms are widely applied in the field of machine vision, but they struggle to perform effectively in detecting positive and negative electrodes on circuit boards, for the following specific reasons:

(1) Circuit boards are left-right symmetric and the positive and negative solder pads are highly similar in morphology. Therefore, the object detection or instance segmentation algorithms based on deep learning can only recognize the positive and negative pad positions from the image, but struggle to distinguish their polarity. Thus, it is difficult to achieve end-to-end detection applying deep learning algorithms, and further processing is still required.
(2) The image acquisition scenario for circuit boards is simple with minimal variations in lighting conditions, traditional machine vision can achieve efficient circuit board segmentation and locating. Deep learning models not only require a large amount of sample images, but also demand huge computing power support for both early training and later operation, and have high hardware performance requirements. Therefore, it is not economical to adopt deep learning algorithms considering development and usage costs.

## 2. Recognition method flow

As can be seen from the image of the buzzer circuit board, the circuit board has the characteristics of left-right symmetry, and the positive and negative solder pads are highly similar in morphology. Moreover, the morphology of the solder pads on different circuit boards is slightly different. The above factors make it difficult to distinguish between the positive and negative polarity based on the morphology of the solder pads. This study proposes a method of transforming image recognition problems into geometric problems based on the geometric characteristics of circuit boards. Geometric analysis is conducted for different orientations of circuit boards to obtain calculation formulas for the positive and negative electrode positions. While identifying the positive and negative electrode positions, the correct distinction between the two polarities is achieved.

**Fig 2:**
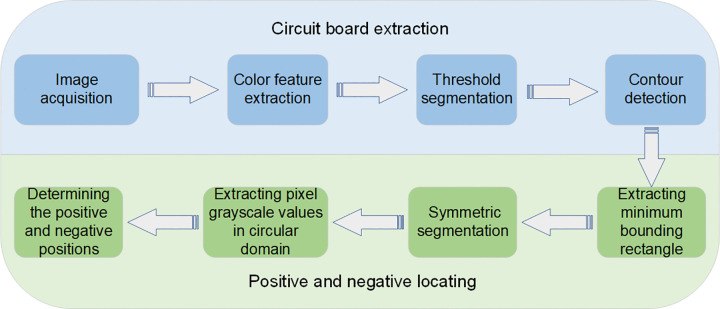
Flow chart of the proposed method.

The flow chart of the proposed method is shown in Fig 2, the method for recognizing positive and negative electrodes of buzzer circuit board is divided into two parts: circuit board extraction and positive and negative locating. Circuit board extraction is mainly based on the color features of the circuit board and combined with filtering, threshold segmentation and contour detection algorithms to separate the circuit board from the image. Positive and negative locating is to approximate the circuit board as a rectangle by calculating the rotated minimum bounding rectangle of each board. And geometric analysis is performed based on this rectangle to determine its longitudinal symmetry axis. Then, by comparing the pixel grayscale values in the central areas on both sides of this longitudinal axis, the side where the positive and negative solder pads are located is determined. Finally, geometric analysis yields a calculation formula for distinguishing between the positive and negative electrode positions of the circuit board in different orientations.

### 2.1. Circuit board extraction

#### 2.1.1. Image acquisition.

**Fig 3:**
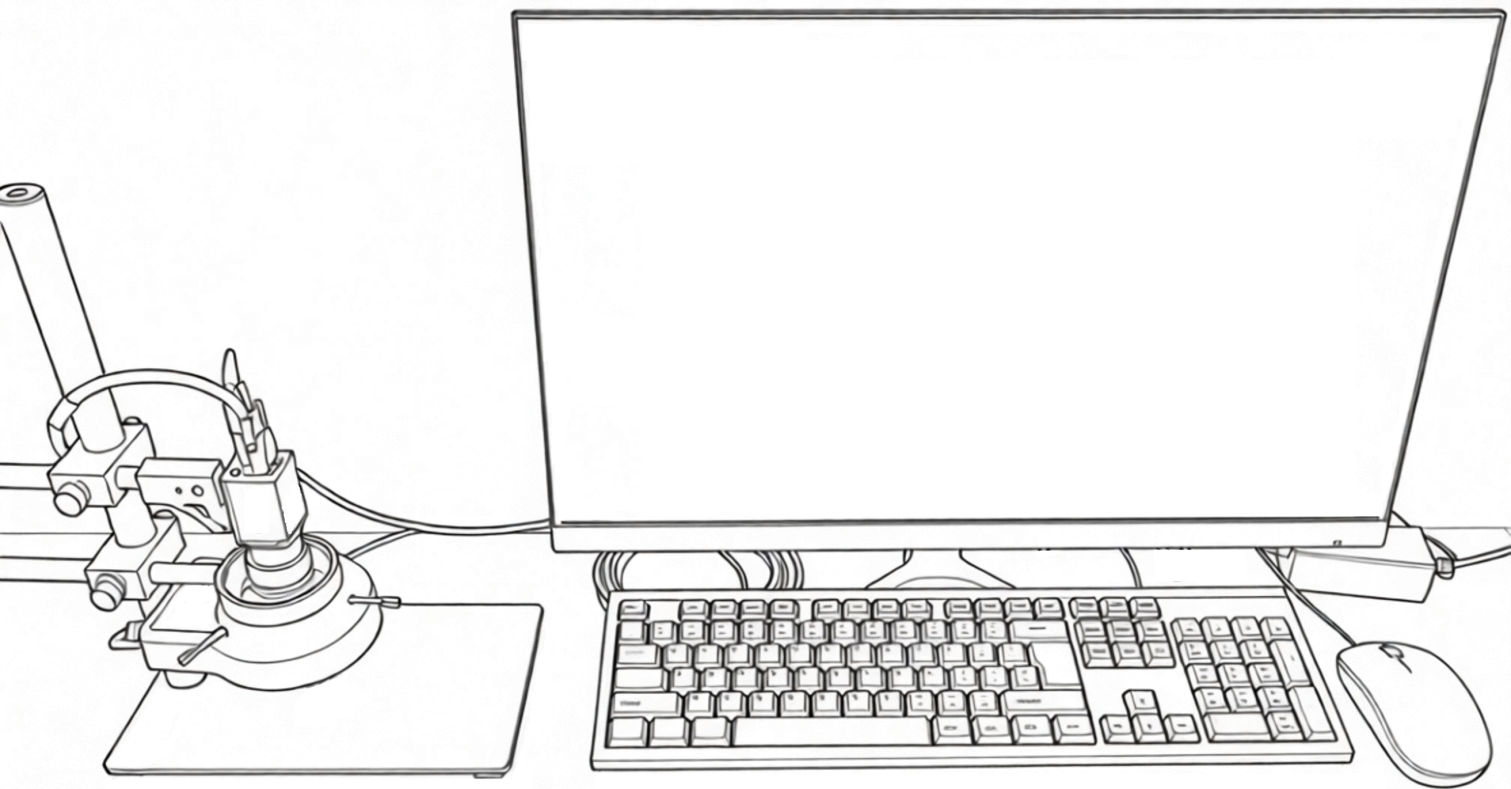
Image acquisition system.

**Fig 4:**
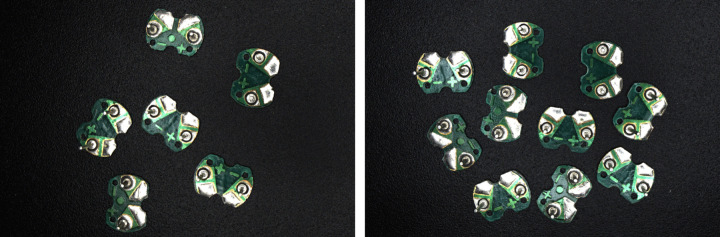
RGB image of the buzzer circuit board.

The image acquisition system consists of a computer, an industrial camera, a lens, a light source and an experimental bench, as shown in Fig 3. The industrial camera selected is Hikrobot MV-CS060–10GC, a 6-megapixel GigE interface area-scan camera equipped with a Sony IMX178 CMOS sensor. It captures images at a resolution of 3072 × 2048 with a 24-bit depth. The focal length of the lens is 8 mm, the aperture is 2.8, and the target surface size is 1/1.8 ″, which is the same as that of the industrial camera. It is connected with the industrial camera by C-mount. A white LED ring light source is used, mounted on the experimental bench to provide high-angle frontal lighting. To facilitate subsequent segmentation of the buzzer circuit board, images are captured with a black tabletop as the background. The acquired RGB image of the buzzer circuit board is shown in Fig 4, where the circuit board occupies approximately 65,000 ± 3,000 pixels. Due to variations in shapes and sizes of the solder pads, the area of the positive and negative solder pads cannot be precisely quantified. Experimental measurements indicate it ranges between 5,000 and 7,000 pixels.

#### 2.1.2. Color feature extraction.

**Fig 5:**
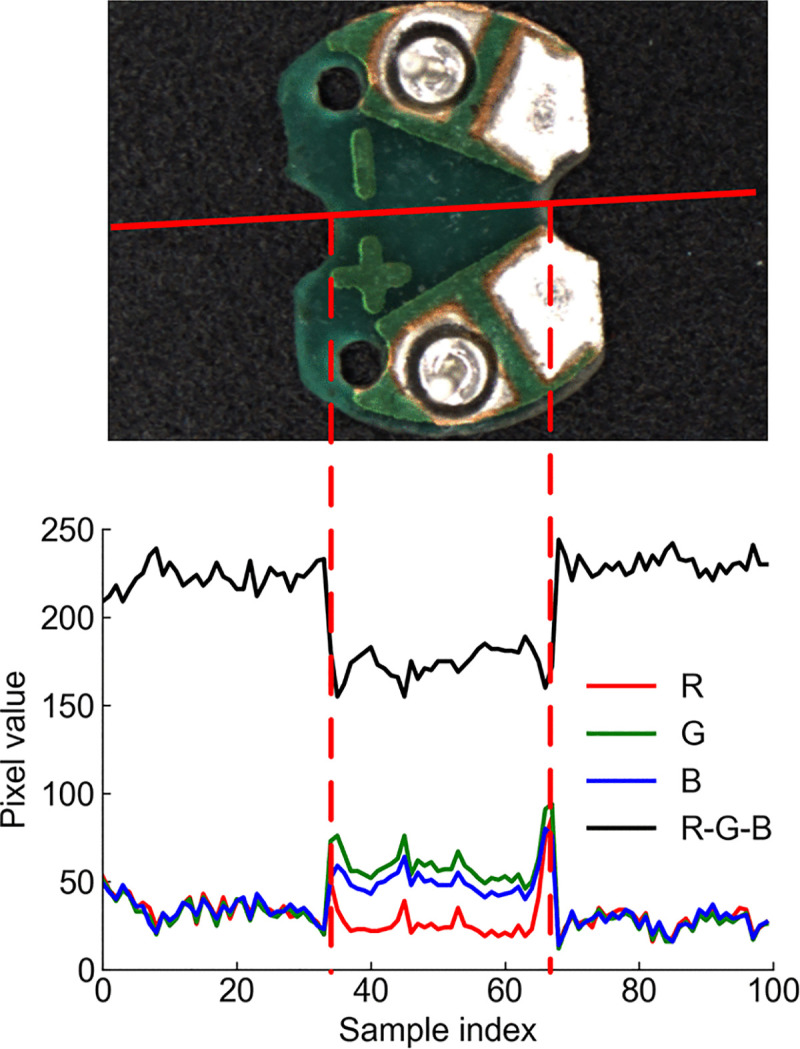
Three channel component and difference curves of the buzzer circuit board.


The circuit board is primarily composed of the substrate and solder pads, appearing visually green and silver-white, while the experimental bench is black. There is a significant difference in color between the two, so color feature extraction is performed first. Fig 5 shows the distribution of the R, G, B channel components along a specific line on the image of buzzer circuit board, as well as the curve representing the difference between the three channels, namely *R-G-B*. Here, the R, G, B channel components are 8-bit unsigned integer data. Considering potential overflow, the subtraction result is actually calculated according to Equation (1). If the value of *R-G-B* is non-negative, the subtraction result is that value; if the value of *R-G-B* is negative, the subtraction result is that value plus 256 to bring it back within the range of 0–255.

$$result=\left\{ \begin{array}{c} @l@R-G-B,R-G-B\geq 0\\ \left(R-G-B\right)+256,R-G-B<0 \end{array}\right.$$ (1)

**Fig 6:**
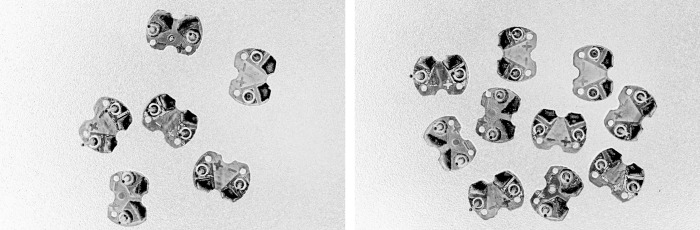
Color difference image.

**Fig 7:**
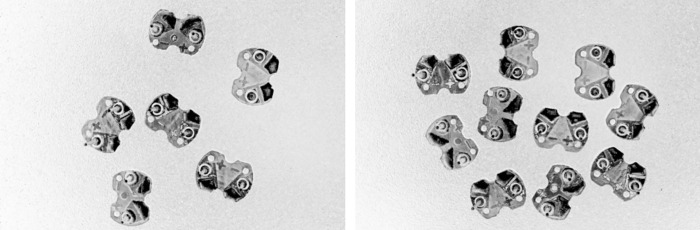
Color difference image after denoising.

As shown in Fig 5, the R, G, and B values of the background along the red solid line are very close, while those of the circuit board are significantly different. By calculating the difference between the three channel components, namely *R-G-B*, a curve is obtained where the pixel values in the middle position show a significant change. The position where this distinct change occurs corresponds to the circuit board region, marked with a red dashed line in the figure. In the subsequent steps, threshold segmentation can be performed using the difference value (*R-G-B*) from the RGB image of the circuit board to distinguish the circuit board from the background. Therefore, the three channel components are first extracted separately and then subtracted to obtain a color difference image, as shown in Fig 6. The color difference image allows for a preliminary distinction between the circuit board and the background: relatively brighter areas represent the background, while relatively darker areas represent the circuit board. However, the color difference image contains a lot of noise. To avoid affecting subsequent processing, denoising is applied directly here. Common denoising algorithms include Gaussian filtering, mean filtering, and median filtering. The noise in the image is not continuously varying along lines but appears as discontinuous point-like artifacts, so Gaussian filtering is not suitable. Between mean filtering and median filtering, experimental tests have shown that mean filtering performs better, using a 7 × 7 convolution kernel. The selection of the filtering kernel size is critical. If the kernel is too small, it causes edge loss on the circuit board; if too large, it blurs the circuit board edges. Both scenarios adversely affect subsequent operations like contour detection, preventing the method from comprehensively and precisely recognizing and detecting the circuit board within the image. The denoised color difference image is shown in Fig 7, with reduced noise and enhanced smoothness compared to the image in Fig 6.

#### 2.1.3. Threshold segmentation.

In order to further simplify the image matrix and increase the distinction between the circuit board and the background, threshold segmentation is performed on the denoised color difference image. Since the circuit board appears darker while the experimental bench appears brighter in the color difference image, an inverse binarization method is specifically selected for threshold segmentation to obtain a white circuit board against a black background. The principle is shown in Equation (2), where g(x,y) represents the pixel value of the output image, f(x,y) represents the pixel value of the input image, and t represents the segmentation threshold.


$$g\left(x,y\right)=\left\{ \begin{array}{c} @l@0,f\left(x,y\right)>t\\ 255,f\left(x,y\right)\leq t \end{array}\right.$$ (2)

**Fig 8:**
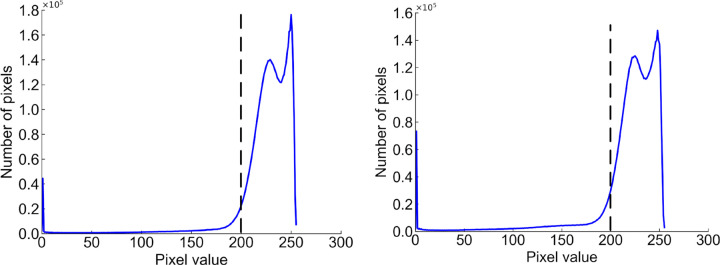
Histogram of the color difference image.

**Fig 9:**
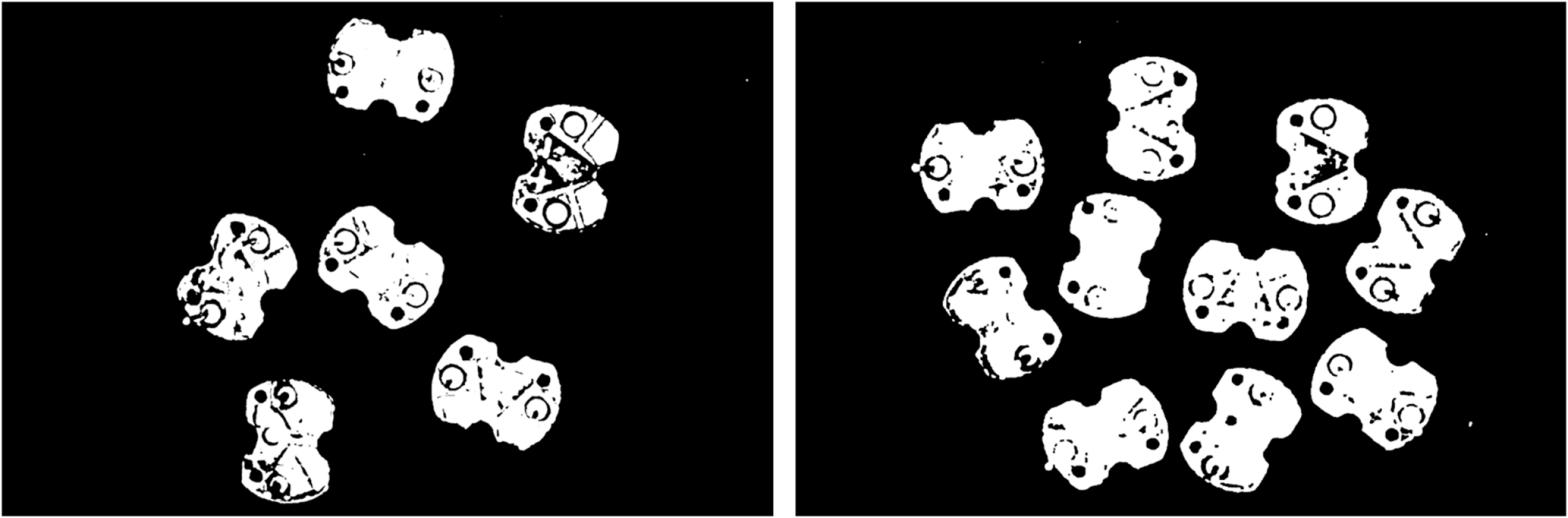
Threshold segmentation image.

**Fig 10:**
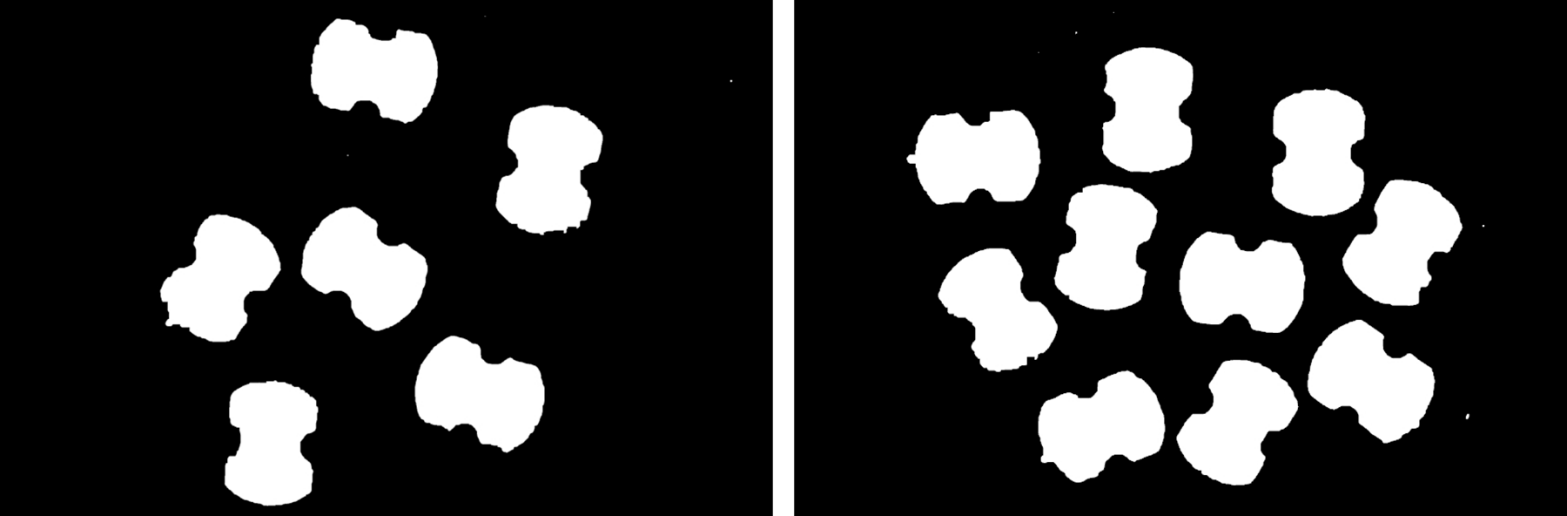
Hole filling image.

From the histogram of the color difference image shown in Fig 8, it can be observed that the curve rises sharply at a pixel value of 200. Using a pixel value of 200 as the boundary, pixels on the left side of the dashed line that are less than 200 correspond to the circuit board region, while pixels on the right side that are greater than 200 correspond to the background region. The threshold here is set at 200, which can separate the circuit board from the background. The result of applying threshold segmentation to the color difference image is shown in Fig 9. From the threshold segmentation result, it can be seen that there are many holes in the circuit board. Therefore, morphological operations such as closing are applied to fill these holes, with the outcome displayed in Fig 10.

#### 2.1.4. Contour detection.

In order to accurately identify all the circuit boards in the image, contour detection is performed on the above preprocessed image. It should be noted that the circuit boards in the preprocessed image may still contain very few holes and noise. To prevent them from being mistakenly detected as contours, the contour detection for circuit boards should be performed in two sequential steps. The first step: Detect contours on the preprocessed image. Only the outermost contour of the circuit board is detected here, ignoring unnecessary internal contours. But in practice, the small holes and noise inside the circuit board may also be detected. The second step: Remove unwanted small contours through the contour area calculation. Since the area of the entire circuit board contour is far larger than that of internal holes or noise contours, contours smaller than the area threshold 5,000 (holes and noise) are removed, while only those larger than the threshold (circuit board) are retained.

### 2.2. Positive and negative locating

#### 2.2.1. Extracting minimum bounding rectangle.

**Fig 11:**
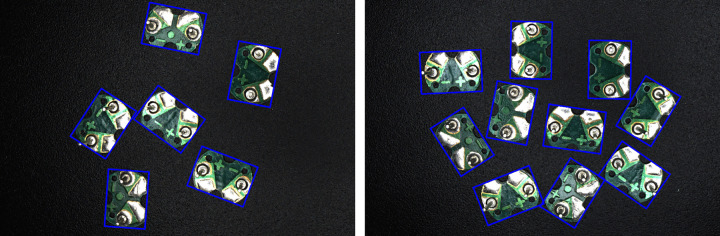
Image of the minimum bounding rectangle.

**Fig 12:**
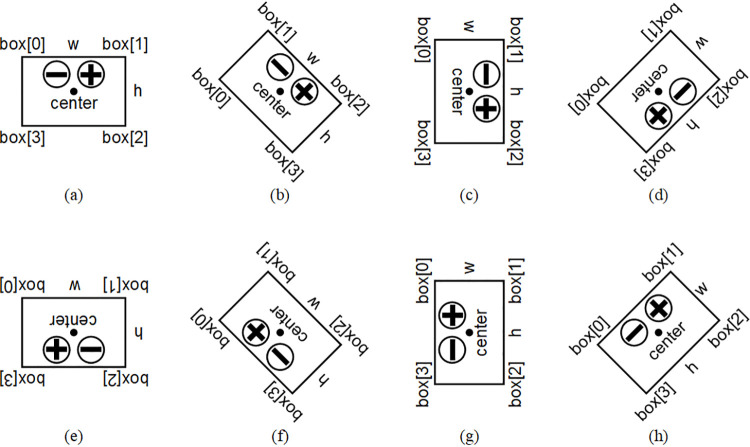
Schematic diagram of the correspondence between the orientation of circuit board and the minimum bounding rectangle with its parameters.

To facilitate geometric analysis of circuit boards, the minimum bounding rectangle is calculated based on the contour information. There are two types of minimum bounding rectangles: one is with rotation angle, the other is without. This paper adopts the former because its rectangular frame is not parallel to the image boundary, making it more fitting to the object than the bounding rectangle without rotation angle. First, calculate the width (w), height (h), center, angle, and vertices (box[0]−box[3]) of the minimum bounding rectangle. Then, draw this minimum bounding rectangle on the original RGB image for ease of observation. The drawing result is shown in Fig 11, where the blue rectangle represents the minimum bounding rectangle of the circuit board. A Cartesian coordinate system is established with the origin at the upper-left corner of the image, the positive x-axis pointing right, and the positive y-axis pointing down. The above parameters are defined by the following rules: Assume a straight line that rotates clockwise around the vertex of the rectangle with the smallest y-coordinate (if y-coordinates are equal, prioritize the smallest x-coordinate), starting from the positive x-axis. The first rectangle side encountered during rotation is defined as w, and the rotation angle at this point is defined as angle, ranging between (0, π/2]. The other side is defined as h. The geometric center of the rectangle is defined as center. Among the four vertices of rectangle, the one with the smallest x-coordinate (if x-coordinates are equal, prioritize the smallest y-coordinate) is labeled box[0], with subsequent vertices labeled clockwise up to box[3]. According to the above rules, there are eight possible cases for the correspondence between the orientation of circuit board and the minimum bounding rectangle with its parameters, as shown in Fig 12. Cases (a), (c), (e), and (g) correspond to the angle of π/2. Cases (b), (d), (f), and (h) correspond to the angle of any value in (0, π/2], which are generally represented between the above cases.

#### 2.2.2. Symmetric segmentation.

**Fig 13:**
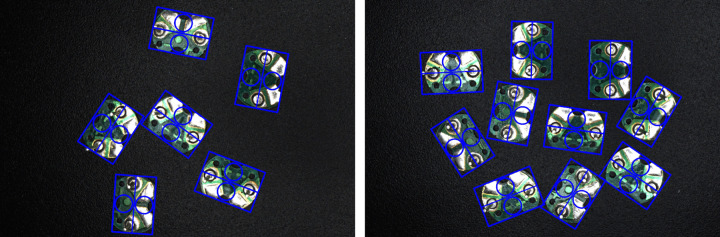
Image of symmetric segmentation and extraction of the circular domain.

**Fig 14:**
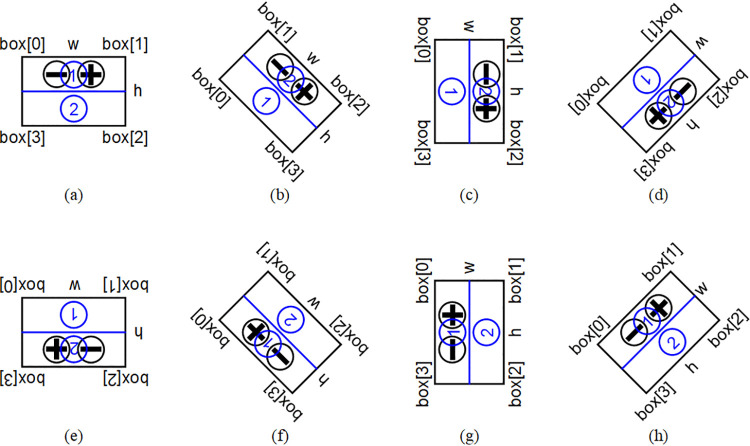
Schematic diagram of the longitudinal symmetry axis and the circular domains for various cases.

Based on the positions of the positive and negative solder pads on the circuit board, it can be observed that the whole board has a geometric characteristic of left-right symmetry and up-down asymmetry. To enable subsequent steps to identify whether the solder pads are located on the upper or lower part of the board, the circuit board is now divided along the longitudinal symmetry axis of its minimum bounding rectangle into upper and lower parts. One contains the solder pads and the other does not, as shown in Fig 13. The blue line in the figure represents the longitudinal symmetry axis, which refers to the line connecting the midpoints of the short sides of the rectangle. However, since the size relationship between w and h of the obtained rectangle is uncertain, it is necessary to compare the sizes of w and h, then consider the smaller one as the short side. For the eight cases mentioned earlier, the two end points of the longitudinal symmetry axis (point1,point2, not distinguished in order) can be calculated according to Table 1. A schematic diagram of the longitudinal symmetry axis for various cases is shown in Fig 14, where the blue line is also employed to represent the longitudinal symmetry axis.

**Table 1:** Two endpoints of the longitudinal symmetry axis (point1, point2) and the two centers of the circular domains (center1, center2).

Case	Is angle π/2	Size between w and h	point1	point2	center1	center2
(a)	Y	w > h	$\frac{box\left[0\right]+box\left[3\right]}{\text{2}}$	$\frac{box\left[1\right]+box\left[2\right]}{\text{2}}$	$\frac{\frac{\text{1}}{\text{2}}\left(box\left[0]+box[1\right]\right)+center}{\text{2}}$	$\frac{\frac{\text{1}}{\text{2}}\left(box\left[2]+box[3\right]\right)+center}{\text{2}}$
(e)						
(d)	N	w < h				
(h)						
(b)	N	w > h	$\frac{box\left[0\right]+box\left[1\right]}{\text{2}}$	$\frac{box\left[2\right]+box\left[3\right]}{\text{2}}$	$\frac{\frac{\text{1}}{\text{2}}\left(box\left[0]+box[3\right]\right)+center}{\text{2}}$	$\frac{\frac{\text{1}}{\text{2}}\left(box\left[1]+box[2\right]\right)+center}{\text{2}}$
(f)						
(c)	Y	w < h				
(g)						

#### 2.2.3. Extracting pixel grayscale values in circular domain.

**Fig 15:**
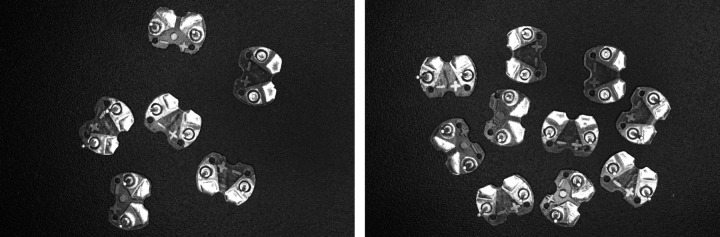
Grayscale image.

In order to identify whether the positive and negative solder pads are located on the upper or lower part of the circuit board, circular domains of equal area are first extracted from symmetric middle positions in both divided parts. Then, the average grayscale values of pixels within these domains (circle1,circle2) are calculated and compared. Because the pixel grayscale values of the positive and negative solder pads are higher than those of the circuit board, circular domains with larger average grayscale values contain the solder pads, while those with smaller averages do not. This paper extracts pixel grayscale values within circular domains on the grayscale image, which is directly converted from the RGB image using the weighted average method, as shown in Fig 15. For ease of observation, the circular domains are drawn on the original RGB image, represented by blue circles in Fig 13. Here, the selection of these two circular domains should be appropriate to ensure that one contains the solder pads while the other does not. The centers of the two circular domains (center1,center2) can be respectively positioned at the midpoints of the lines connecting the midpoints of the two long sides of the minimum bounding rectangle and the center of that rectangle (center). For the eight cases mentioned earlier, the specific positions of center1,center2 are detailed in Table 1, which are approximately located in the middle of the two divided parts. As for the radius of the two circular domains, the values should not be too small, so that one of the circular domains contains positive and negative solder pads. However, the radius should also not be excessively large, to avoid increasing the subsequent calculation of grayscale values and introducing interference from irrelevant areas. Through experimental testing, a radius of 50 pixels for the two circular domains has proven reasonable. This achieves the effect shown in Fig 13, where one circular domain precisely encloses the solder pads while the other is symmetrically located on the opposite part. Schematic diagram of circular domains for various cases is drawn in Fig 14, where blue circles are also employed to represent the drawn circular domains.

#### 2.2.4. Determining the positive and negative positions.

**Fig 16:**
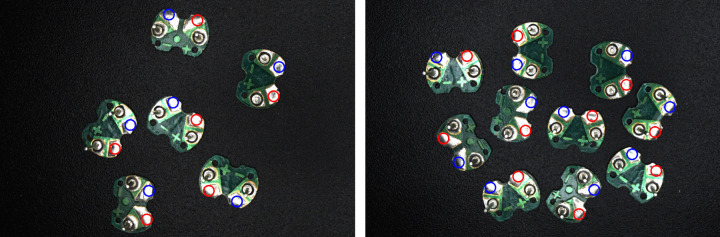
Image of the positive and negative recognition result.

Based on the minimum bounding rectangle and its parameters, specifically whether the angle is π/2 and the relationship between w and h, the eight cases (a) to (h) in Fig 12 can be categorized into four groups: (a) and (e), (b) and (f), (c) and (g), (d) and (h). For the two cases within each group, the orientation and parametric characteristics of minimum bounding rectangles are exactly the same, only the positions of the positive and negative electrodes (c1,c2) are different. These positions can be distinguished by comparing the average grayscale values (circle1,circle2) of the pixels within the two circular domains. Once the specific case is determined, c1 and c2 are determined accordingly. The logic and calculation method for determining c1 and c2 are detailed in Table 2. For ease of observation, positive and negative solder pads are marked with circles centered at c1 and c2 in the original RGB image, as shown in Fig 16. In the figure, the red circle is employed to mark the positive solder pad, and the blue circle is employed to mark the negative solder pad.

**Table 2:** Positive and negative positions (c1, c2).

Is angle π/2	Size between w and h	Size between circle1 and circle2	Case	c1	c2
Y	w > h	circle1 > circle2	(a)	$\frac{box\left[1\right]+center1}{\text{2}}$	$\frac{box\left[0\right]+center1}{\text{2}}$
		circle1 < circle2	(e)	$\frac{box\left[3\right]+center2}{\text{2}}$	$\frac{box\left[2\right]+center2}{\text{2}}$
N	w > h	circle1 < circle2	(b)	$\frac{box\left[2\right]+center2}{\text{2}}$	$\frac{box\left[1\right]+center2}{\text{2}}$
		circle1 > circle2	(f)	$\frac{box\left[0\right]+center1}{\text{2}}$	$\frac{box\left[3\right]+center1}{\text{2}}$
Y	w < h	circle1 < circle2	(c)	$\frac{box\left[2\right]+center2}{\text{2}}$	$\frac{box\left[1\right]+center2}{\text{2}}$
		circle1 > circle2	(g)	$\frac{box\left[0\right]+center1}{\text{2}}$	$\frac{box\left[3\right]+center1}{\text{2}}$
N	w < h	circle1 < circle2	(d)	$\frac{box\left[3\right]+center2}{\text{2}}$	$\frac{box\left[2\right]+center2}{\text{2}}$
		circle1 > circle2	(h)	$\frac{box\left[1\right]+center1}{\text{2}}$	$\frac{box\left[0\right]+center1}{\text{2}}$

## 3. Experiments and analyses

### 3.1. Experimental platform

The computer processor is Intel i5-10400, the memory is 16GB, and the hard drive is 1TB SSD. The operating system running on this computer is Windows 10, 64-bit edition. The programming language is Python, the editor uses PyCharm, and the support library uses the open-source and cross-platform computer vision library OpenCV.

### 3.2. Experiments and analyses of results

#### 3.2.1. Experiment and result analysis of extracting the minimum bounding rectangle.

As can be seen from the aforementioned method flow, both geometric analysis and the calculation of positive and negative electrode positions are based on the extracted minimum bounding rectangle. Therefore, whether the minimum bounding rectangle can be accurately extracted directly affects the accuracy of the final positive and negative electrode recognition and locating. In this paper, a total of 30 circuit boards are randomly selected as samples from the captured images to conduct the experiment of extracting the minimum bounding rectangle. On the one hand, the circuit boards in the samples are preprocessed by the proposed method to extract the minimum bounding rectangle, and the data obtained are treated as the experimental values. On the other hand, the circuit boards in the samples are annotated at the pixel level with the help of a high-precision image annotation tool, which replaces the preprocessing steps in the method. Then, the extraction of the minimum bounding rectangle is conducted, and the data obtained are treated as the standard values. The error in extracting the minimum bounding rectangle can be obtained by comparing the experimental values with the standard values.

**Fig 17:**
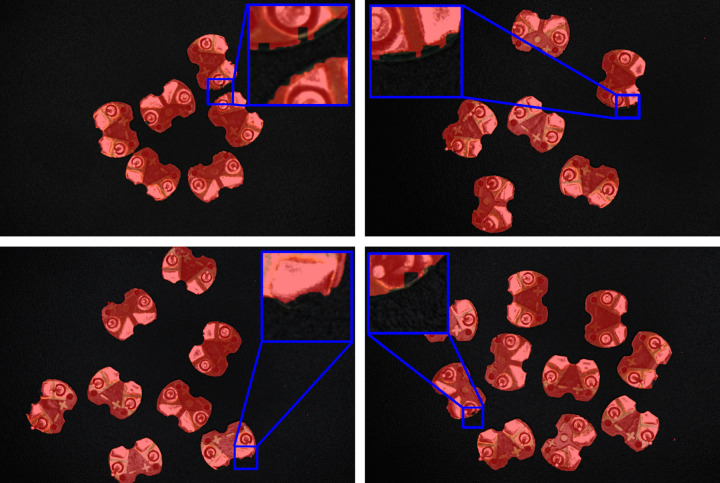
Binary image obtained by the method overlaid on the original RGB image.

**Fig 18:**
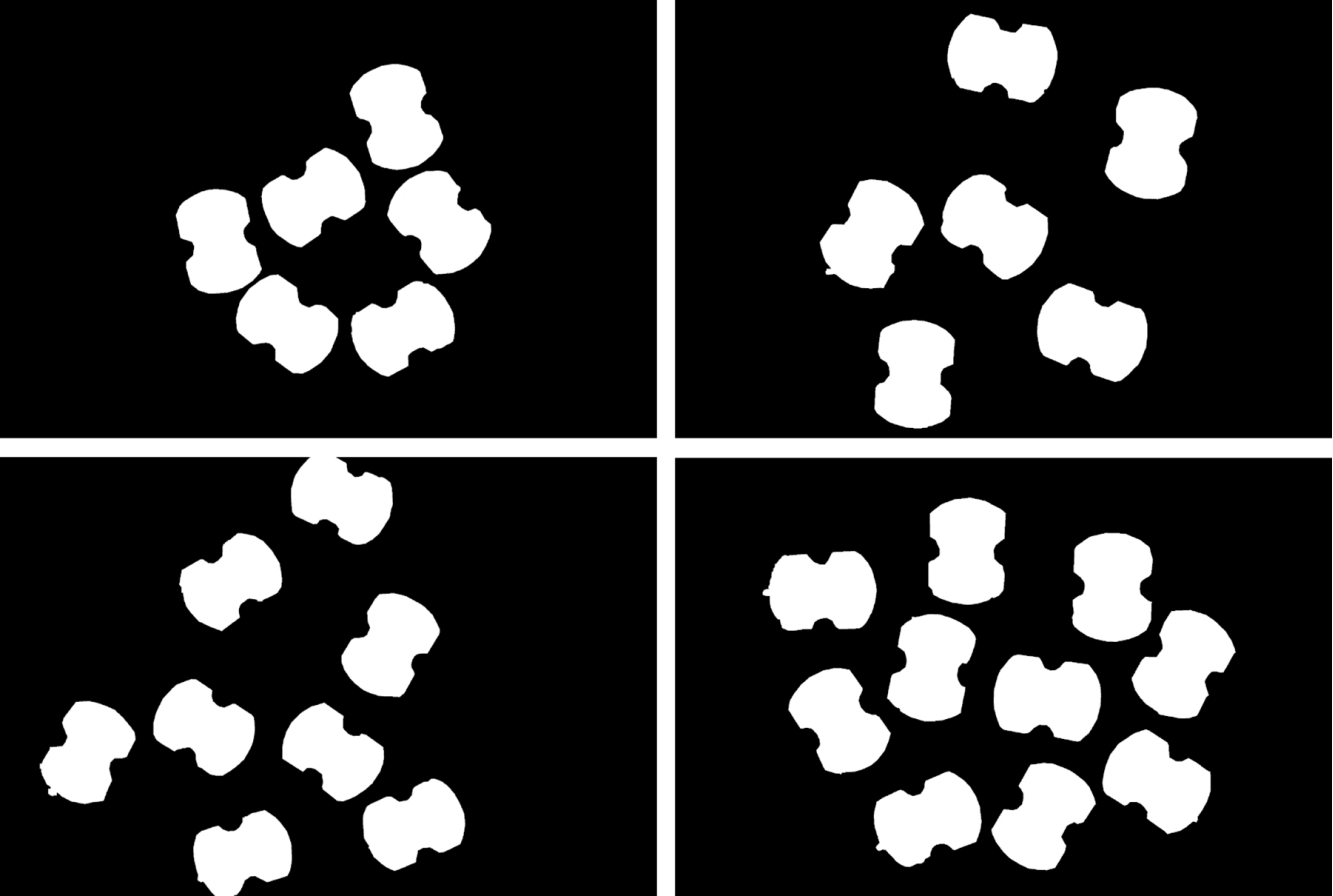
Binary image obtained by annotating the circuit board.

The binary image of the circuit board obtained by the method is now overlaid on the original RGB image, as shown in Fig 17. In the figure, the red area represents the binary image of the circuit board, and the blue box represents certain areas of the circuit board along with their locally magnified images. As can be seen from the figure, the binary image of the circuit board almost coincides with the original RGB image, with only minor deviations at some edge positions. Therefore, it can be anticipated that the error in subsequently extracting the minimum bounding rectangle will be small. The binary image obtained by pixel level annotation of the circuit board using the image annotation tool is shown in Fig 18. It is evident that the annotation precision of this tool is very high, so the data of minimum bounding rectangle extracted subsequently can be regarded as reference standard values.

**Fig 19:**
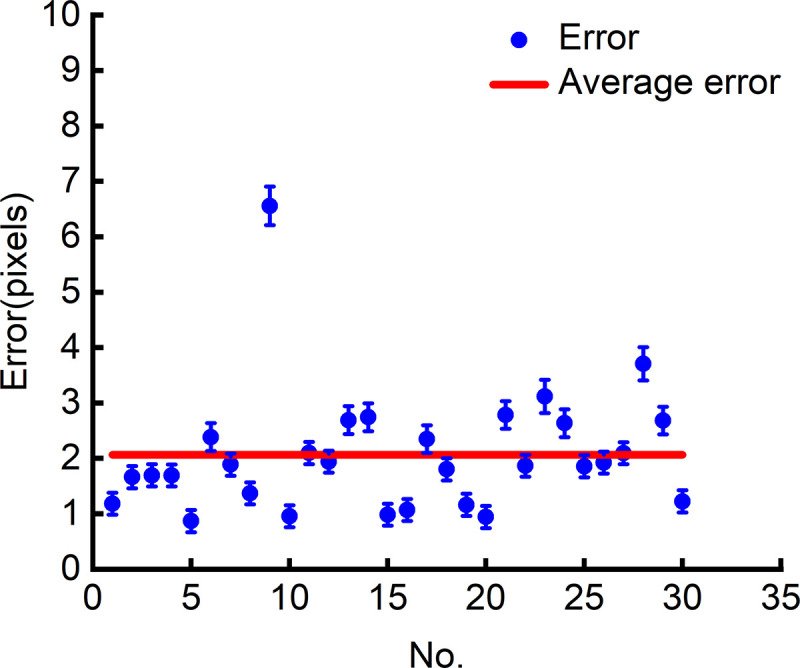
Error of the parameter center.

**Fig 20:**
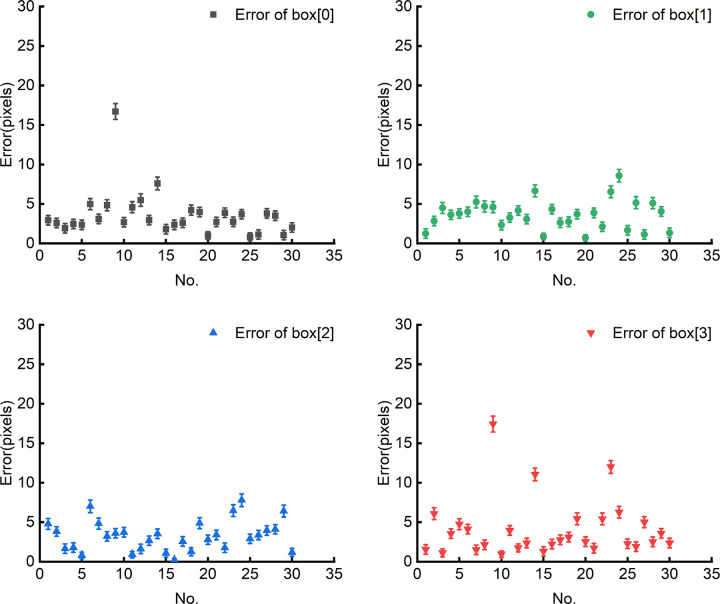
Errors of the parameters box[0]–box[3].

Because the parameters of the minimum bounding rectangle are highly coupled, once certain parameters are determined, the others are also determined accordingly. Therefore, the paper only analyses the errors of the parameterscenterand box[0]−box[3]. The errors in parameters center and box[0]−box[3] also indirectly reflects the errors in other parameters angle, w and h, demonstrating the accuracy of extracting the minimum bounding rectangle. The error of the parameter center is shown in Fig 19, where the blue scatter points represent the error of the respective parameter center for 30 circuit boards from number 1 to number 30, and the red solid line represents the average error. The errors of parameters box[0]−box[3] are shown in Fig 20, where black, green, blue and red scatter points represent the errors of parameters box[0], box[1], box[2]and box[3], respectively. The errors of these parameters are all characterized by Euclidean distance in pixels.

As shown in Fig 19, the error of the minimum bounding rectangle parameter centerremains below 10 pixels, with an average error of only about 2 pixels. As shown in Fig 20, the errors of the minimum bounding rectangle parameters box[0]−box[3] are almost all below 10 pixels. On an image with a resolution of 3072 × 2048, the error in extracting the minimum bounding rectangle is only single-digit(zero to ten) pixel values, indicating that the method proposed in this paper demonstrates good performance during the image preprocessing stage.

#### 3.2.2. Experiment and result analysis of positive and negative recognition.

**Fig 21:**
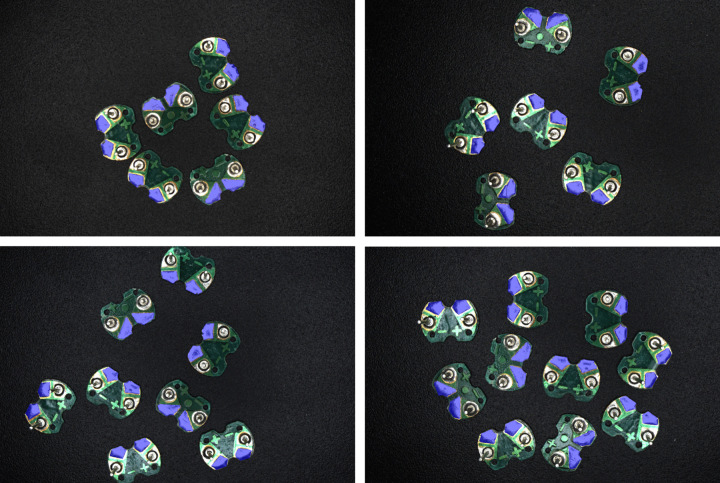
Process image of annotating the positive and negative electrodes.

**Fig 22:**
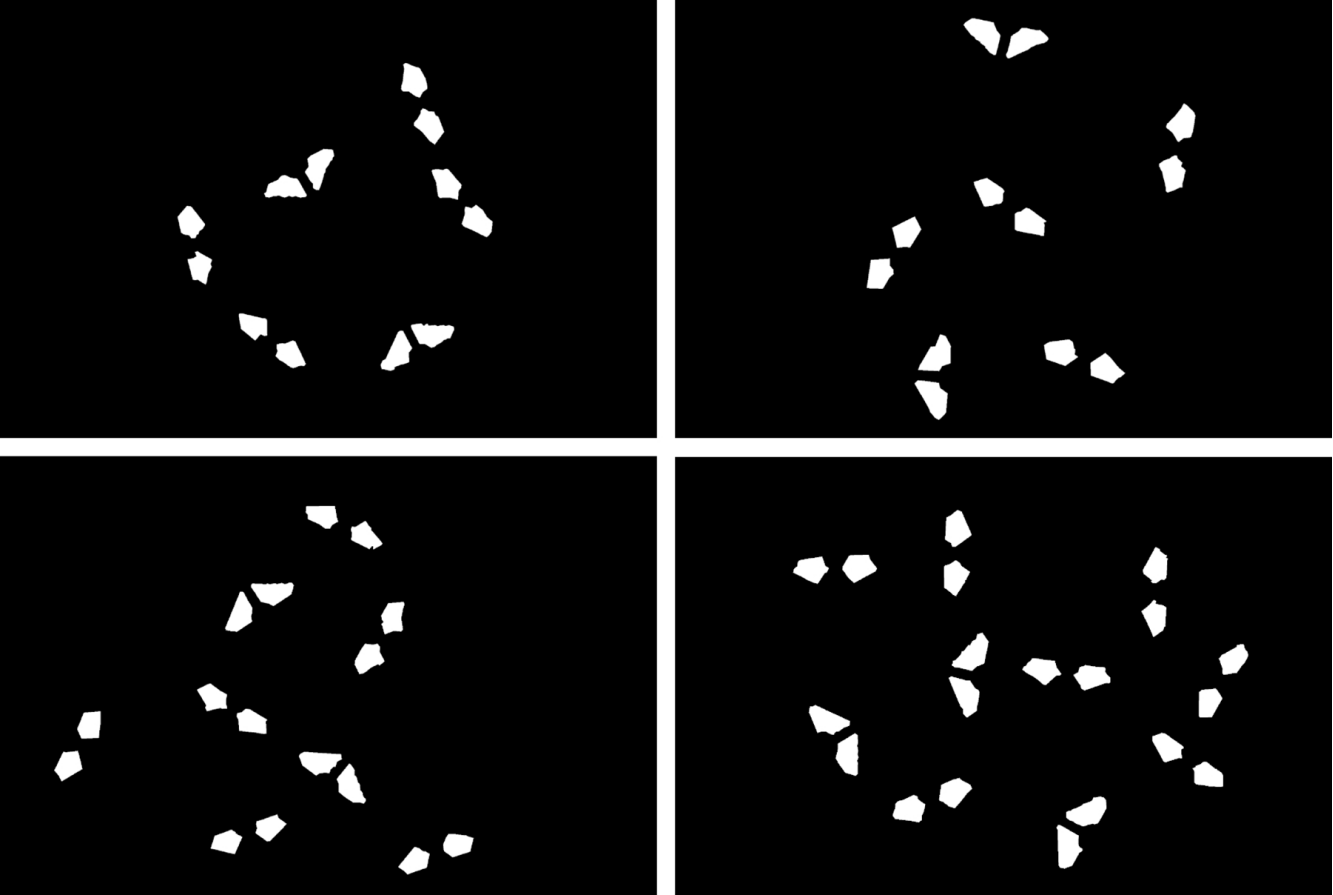
Binary image obtained by annotating the positive and negative electrodes.

In order to test the final recognition accuracy of the proposed method, the same 30 circuit board samples from the previous experiment were still used as experimental subjects, containing a total of 60 positive and negative electrodes. On the one hand, the positive and negative electrode positions are calculated by the proposed method, and the data obtained are treated as the experimental values. On the other hand, the positive and negative electrodes of circuit boards are annotated at the pixel level with the help of a high-precision image annotation tool. Then, connected component analysis is conducted. The center of the annotated areas are the positive and negative electrode positions, and the data obtained are treated as the standard values. The final recognition error of the proposed method can be obtained by comparing the experimental values with the standard values. The process of pixel level annotation of the positive and negative electrodes using the image annotation tool is shown in Fig 21, and the resulting binary image is shown in Fig 22.

In fact, the positions of the positive and negative electrodes recognized by the proposed method are represented by the center coordinates of the corresponding pads, which are used as the experimental values in the analysis. Through manual marking, both the center coordinates and the area of the pads can be obtained. While the center coordinates serve as the experimental values, the pad area data cannot be directly obtained by the proposed method. Now the existing data is as follows: the experimentally obtained center coordinates of the positive and negative solder pads from the proposed method, the manually marked center coordinates of the positive and negative solder pads and the manually marked area of the positive and negative solder pads. In this study, the manually marked center coordinates are selected as the standard values. Then the product of the difference in the coordinate components between the experimental and standard values is calculated, yielding a quantity with the dimension of area. The manually marked area of the positive and negative solder pads is selected as the benchmark and reference because the degree of overlap between the recognized pads and the actual pads intuitively reflects the accuracy of the proposed method. This overlap is directly related to potential anomalies in actual soldering processes, such as poor soldering between solder joints and corresponding positive and negative pads. Ultimately, Equation (3) is derived, where the error is characterized by the ratio of the product of the coordinate component deviations between the experimental and standard values of the positive and negative electrode positions to the pad area.


$$e=\frac{\Delta x\Delta y}{S}\times 100\%$$ (3)

**Fig 23:**
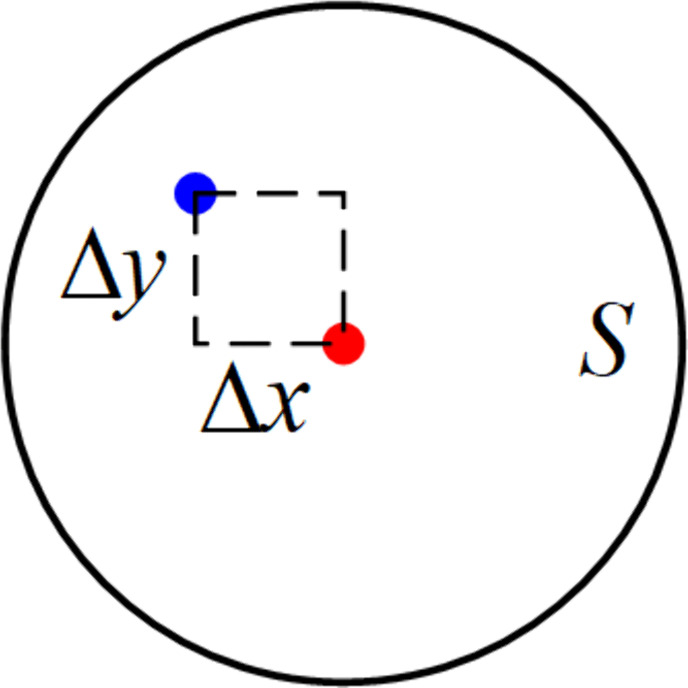
Schematic diagram of the parameters about recognition result.

Here, Δx represents the horizontal coordinate deviation (always taken as positive) between the experimental and standard values of the positive and negative positions, Δy represents the vertical coordinate deviation (always taken as positive) between the experimental and standard values of the positive and negative positions, S represents the area of the positive and negative electrodes, and e is the relative error. The schematic diagram of the above-mentioned parameters is provided in Fig 23.

**Fig 24:**
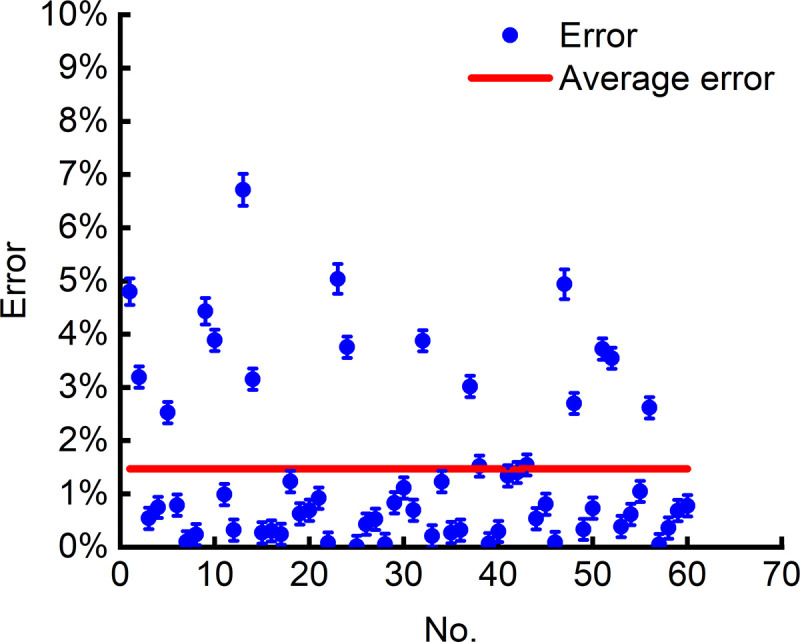
Error in recognizing the positive and negative electrode positions.

The error in recognizing the positive and negative electrode positions is shown in Fig 24, where the blue scatter points represent the relative errors of the respective positions of a total of 60 positive and negative electrodes on 30 circuit boards, and the red solid line represents the average relative error. As seen in Fig 24, the relative errors in recognizing the positive and negative electrode positions are mostly below 4%, with an average relative error of less than 2%. This demonstrates that the method proposed in this paper achieves robust recognition and locating performance.

#### 3.2.3. Comparison and analysis of different methods.

Due to the absence of directly comparable research—specifically on the recognition of positive and negative electrodes of buzzer circuit boards, this paper selects methods related to other applications of machine vision in the electronics industry mentioned in the introduction, and compares and analyzes them with the method proposed in this study. The specific details are presented in Table 3. The application areas include Mark point positioning, solder joint recognition, and defect detection on circuit boards. The methods compared range from traditional image processing to modern machine learning and deep learning-based approaches, with accuracy serving as the evaluation metric. As can be seen from Table 3, when compared with various methods from different application areas based on machine vision, the recognition accuracy of the method proposed in this paper is higher than that of most methods, second only to the methods proposed in references [18] and [25], reaching 98.6%. This indicates that the proposed method possesses good recognition performance and practical application significance.

**Table 3:** Performance comparison of different methods.

Reference	Area	Method	Accuracy
[16]	Mark point positioning	improved Hough transform	98.5%
[17]	Mark point positioning	IBBS-SIFT	91.0%
[18]	Mark point positioning	sub-pixel least squares fitting	99.6%
[19]	solder joint recognition	SIFT with edge detection	95.0%
[21]	solder joint recognition	improved YOLOv4	95.6%
[22]	solder joint recognition	circular fitting similarity calculation	93.0%
[24]	defect detection	background feature probability calculation	94.9%
[25]	defect detection	PSO, FLANCC and RANSAC	98.9%
[28]	defect detection	Mars-Net and HVP	84.7%
[29]	defect detection	EMRA-Net	95.3%
[30]	defect detection	improved YOLOv5	98.5%
[31]	defect detection	PCB-YOLO	96.3%
[33]	defect detection	CM-YOLO	96.9%
[34]	defect detection	improved YOLO11n	95.0%
Proposed	electrode recognition	/	98.6%

## 4. Conclusion

In order to automate the soldering of coils to circuit boards in buzzers, this study proposes a method for recognizing positive and negative electrodes of buzzer circuit board based on machine vision. The method first employs the color difference operator *R-G-B* to extract the color feature map of the circuit board. Next, algorithms such as filtering, threshold segmentation and contour detection are employed to extract the circuit board contours, and the rotated minimum bounding rectangle of each circuit board is obtained to achieve precise locating. Then, according to the up-down asymmetric geometric characteristics of the circuit boards, the positive and negative electrode recognition problem is converted into a simple geometric analysis problem. According to the current orientation of the circuit board, it is categorized into eight cases, and corresponding calculation formulas of positive and negative position are designed, effectively distinguishing and locating the positive and negative electrodes of the circuit board. Experimental results demonstrate high recognition accuracy of the method: The error in extracting the minimum bounding rectangle is only single-digit(zero to ten) pixel values. Finally, the average relative error in recognizing the positive and negative electrode positions is less than 2%. After testing, the method proposed in this paper demonstrates effective recognition and locating performance. It holds practical value for production, and is expected to be applied to automatic soldering equipment to achieve automated soldering of buzzer circuit boards.

This method is developed and evaluated specifically for buzzer circuit boards, but it also possesses a certain degree of generality. It can be extended to recognize the positive and negative electrodes and solder pads on other types of circuit boards. For instance, components such as electrolytic capacitors, infrared receiver modules, negative temperature coefficient sensors, CdS photoresistors, Hall switches, vibration motors, and wireless charging receiver coils also require electrode recognition during soldering onto circuit boards. The solder pads corresponding to these components on the boards are similar in position and appearance to those on the buzzer circuit boards. Their pads are geometrically symmetric and positioned at the top of the board, visually appear silver white in color, and the circuit board substrate is typically blue and green. Therefore, the method for recognizing positive and negative electrodes of buzzer circuit board based on machine vision proposed in this paper can also be applied to the electrode and solder pad recognition of circuit boards for the aforementioned components.
